# Mapping the functional anatomy and topography of the cardiac autonomic innervation for selective cardiac neuromodulation using MicroCT

**DOI:** 10.3389/fcell.2022.968870

**Published:** 2022-09-12

**Authors:** Bettina Kronsteiner, Lydia M. Zopf, Patrick Heimel, Gunpreet Oberoi, Anne M. Kramer, Paul Slezak, Wolfgang J. Weninger, Bruno K. Podesser, Attila Kiss, Francesco Moscato

**Affiliations:** ^1^ Center for Medical Physics and Biomedical Engineering, Medical University of Vienna, Vienna, Austria; ^2^ Ludwig Boltzmann Institute for Cardiovascular Research, Vienna, Austria; ^3^ AUVA Research Centre, Ludwig Boltzmann Institute for Experimental and Clinical Traumatology, Vienna, Austria; ^4^ Austrian Cluster for Tissue Regeneration, Vienna, Austria; ^5^ Karl Donath Laboratory for Hard Tissue and Biomaterial Research, University Dental Clinic Vienna, Vienna, Austria; ^6^ Department of Anatomy, Center for Anatomy and Cell Biology, Medical University of Vienna, Vienna, Austria; ^7^ Division of Anatomy, Center for Anatomy and Cell Biology, Medical University of Vienna, Vienna, Austria

**Keywords:** vagus nerve stimulation, cardiovascular diseases, fascicular anatomy, selective cardiac neuromodulation, microcomputed tomography, 3D rendering Kronsteiner et al. imaging of cardiac autonomic innervation

## Abstract

**Background:** Vagus nerve stimulation (VNS) has gained great importance as a promising therapy for a myriad of diseases. Of particular interest is the therapy of cardiovascular diseases, such as heart failure or atrial fibrillation using selective cardiac VNS. However, there is still a lack of organ-specific anatomical knowledge about the fascicular anatomy and topography of the cardiac branch (CB), which diminishes the therapeutic possibilities for selective cardiac neuromodulation. Here, we established a topographical and anatomical map of the superior cardiac VN in two animal species to dissect cervical and cardiac VN morphology.

**Methods:** Autonomic nerves including superior CBs were harvested from domestic pigs and New Zeeland rabbits followed by imaging with microcomputed tomography (µCT) and 3D rendering. The data were analyzed in terms of relevant topographical and anatomical parameters.

**Results:** Our data showed that cardiac vagal fascicles remained separated from other VN fascicles up to 22.19 mm (IQR 14.02–41.30 mm) in pigs and 7.68 mm (IQR 4.06–12.77 mm) in rabbits from the CB point and then started merging with other fascicles. Exchanges of nerve fascicles between sympathetic trunk (ST) and VN were observed in 3 out of 11 nerves, which might cause additional unwanted effects in unselective VNS. Our 3D rendered digital model of the cardiac fascicles was generated showing that CB first remained on the medial side where it branched off the VN, as also shown in the µCT data of 11 pig nerves, and then migrated towards the ventromedial site the further it was traced cranially.

**Conclusion:** Our data provided an anatomical map of the cardiac vagal branches including cervical VN and ST for future approaches of selective cardiac neurostimulation, indicating the best position of selective cardiac VNS just above the CB point.

## 1 Introduction

The autonomic nervous system (ANS) plays a critical role in the subconscious functional control of numerous organs under physiological and pathological conditions [(Benarroch, 2020), (Vilensky et al., 2015)]. The two responsible key players of ANS, the sympathetic and the parasympathetic nervous systems, regulate physiological organ functions and maintain homeostasis. However, the imbalance between sympathetic and parasympathetic projections has been extensively studied and it was found to be associated with organ malfunction, inflammation, and cardio- and cerebrovascular diseases [(Tubbs et al., 2004; Awad et al., 2016; Bonaz et al., 2018; Kohoutova et al., 2019)].

The main branch of the parasympathetic nervous system, the vagus nerve (VN), innervates numerous organs throughout the body, thereby maintaining the so-called “rest and digest” situations, whereas its antagonistic nerve, the sympathetic trunk (ST) acts with “fight or flight” responses [(Wink et al., 2020; Wehrwein et al., 2016)]. In the cardiovascular system, VN is known to be important for the adaptation and regulation of heart rate and blood pressure. To date, the most common therapies applied to various diseases and pathologies are pharmacological and surgical interventions [(Mishra, 2017)]. However, novel neuromodulation strategies of peripheral nerves, such as VN, could show positive results towards replacement or at least reduction of extensive use of pharmacological drugs, which have caused adverse effects in patients. Recent studies demonstrated the potential benefit of VNS, e.g., acute or chronic VNS was able to improve the burden of atrial fibrillation, severity of rheumatoid arthritis, pain, or mitigation of inflammation [(Tubbs et al., 2004; Bonaz et al., 2018; Kohoutova et al., 2019; Brack et al., 2011; De Ferrari and Schwartz, 2011; Johnson and Wilson, 2018)]. Therefore, VNS is the prime target of numerous clinical [(Ter Horst et al., 1996)] and therapeutic approaches, such as depression [(Grimonprez et al., 2015)], rheumatoid arthritis [(Koopman et al., 2016)], epilepsy [(Alexopoulos et al., 2006), (Chambers and Bowen, 2013)], and immunoregulatory diseases [(Pavlov et al., 2003)]. In cardiovascular medicine, VNS has already been shown to mitigate heart damage caused by pathologies such as atrial fibrillation or ischemic infarct [(Brack et al., 2011; Sheng et al., 2011; Gao et al., 2015; Nuntaphum et al., 2018; Capilupi et al., 2019; Thompson et al., 2020a)]. Nevertheless, large randomized clinical trials failed to prove the efficacy of VNS that was applied among patients with heart failure [(Gold et al., 2016)]. Of importance to emphasize in these previous studies, VNS was dominantly applied via cuff electrodes around VN at the cervical levels. However, there is accumulating evidence that due to the complex anatomy of cervical VN, innervating almost all organs of the body, the unselective stimulation of the cervical VN inevitably leads to off-target effects by the stimulation of unintended nerve fascicles [11]. The purpose of this study was to map the topography and anatomy of the cardiac vagal autonomic innervation of rabbits and pigs from the cervical level down to the heart using µCT imaging and 3D reconstructions. In addition, anatomical key features such as fascicle numbers as well as the size and area of the nerve were quantitatively measured at different levels.

## 2 Materials and methods

### 2.1 Surgical dissection and macroscopic examination of nerve samples

**FIGURE 1:**
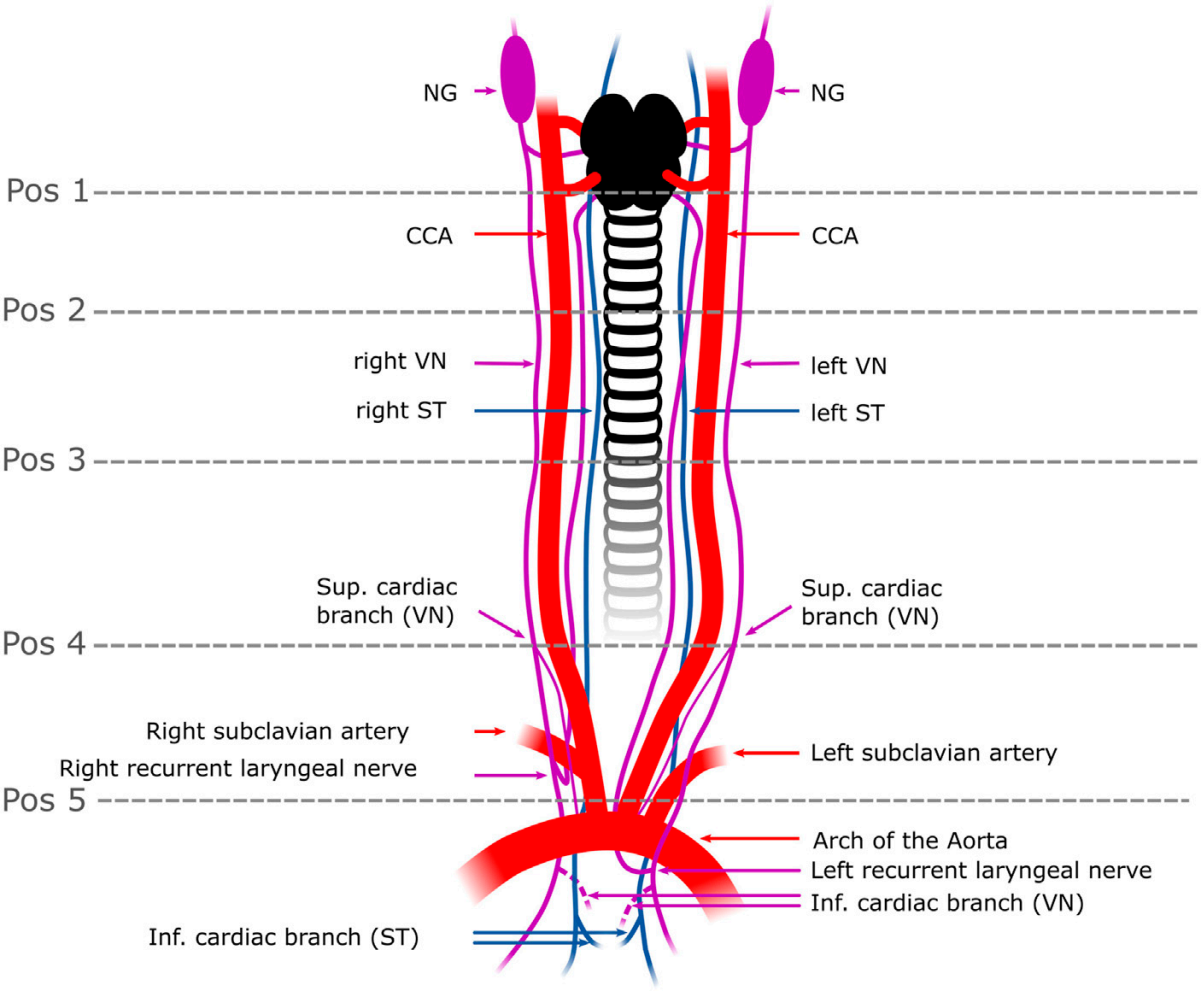
Schematic overview of the surgical window in a pig. The VN can be distinguished from the ST by identification of the NG. In some individuals, the two nerves were observed to merge or split at the cervical level. In rabbits, the VN and the ST were travelling individually next to each other to the cardiac plexus. Five anatomical positions (pos 1-pos 5) were defined from cranial to caudal at which the fascicle number and nerve areas were measured. The superior CB was the branch used in this study (one superior CB per nerve) and harvested right above the level of the subclavian artery (pos.5); the inferior CB (indicated as dashed lines) was not used in this study since its anatomical position in the epicardial fat is surgically less feasible for selective stimulation than the superior CB. Pos. 1-3 label the upper to the mid-cervical level. Pos. 4 indicates the cardiac branching point of the superior CB. NG, nodose ganglion; CCA, common carotid artery; VN, Vagus Nerve; ST, Sympathetic trunk; Pos, position; sup. cardiac branch, superior cardiac branch; inf. cardiac branch, inferior cardiac branch.

Fresh cadavers of male (n = 3) and female (n = 4) pigs (domestic pigs, n = 7, nerve harvest 1–2 h post mortem (p.m.)., 36–64 kg, age of 6 months) as well as female rabbits (New Zealand White, n = 3, nerve harvest 1–2 h p.m., 2.5–3.3 kg body weight, age of 3–4 months) were donated immediately after euthanasia for dissection and sampling of fresh autonomic nerve specimen including superior cardiac vagal branches (3R principle). All experimental procedures were ethically reviewed and approved by the Ethics Committee for Laboratory Animal Research of the Medical University of Vienna (**pigs:** GZ 2021-0.615.897//**rabbits:** 0030-V/3b/2018) and of the Austrian Federal Ministry of Science, Research, and Economy. Animals were treated in accordance with the “Guide for the Care and use of Laboratory Animals” (NIH publication 85–23, revised 1985). Out of all cadavers (n = 7 pigs, n = 3 rabbits) given to our study, 11 pig nerves (n = 5 left, n = 6 right nerves) and 5 rabbit nerves (n = 2 left nerves, n = 3 right nerves) could be retrieved for use in this study. A schematic diagram of the surgical field was created using the vector graphics tool Inkscape (Inkscape, 2016) as shown in Figure 1. In pigs, the nerves were dissected and harvested as is depicted in Figure 1, demonstrating that the superior cardiac branch was only dissected. A similar surgical window was used in rabbits as well.

**FIGURE 2:**
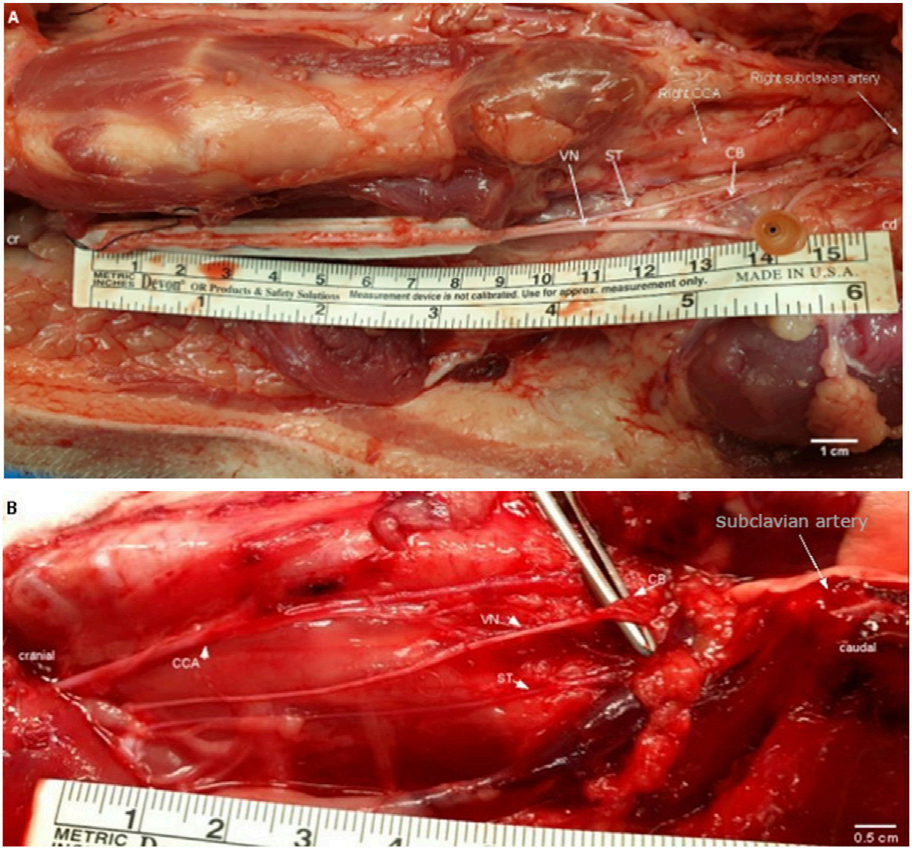
**(A)**. Surgical window of the right cervical and cardiac region in a pig. White arrows point at the VN, the ST, and the superior CB. The positions of the CCA and right subclavian artery are labeled for anatomical orientation. The inferior cardiac branch was not used in this study and is located caudal to the subclavian artery. Cr, cranial; cd, caudal; CCA, common carotid artery; VN, cervical vagus nerve; ST, sympathetic trunk; CB, superior cardiac branch. Cr, cranial; cd, caudal; CCA, common carotid artery; VN, cervical vagus nerve; ST, sympathetic trunk; CB, superior cardiac branch. **(B)**. Surgical window of the right cervical and cardiac region in a rabbit. The cervical VN gives one superior CB to the heart. White arrows point at the VN; the ST; and the superior CB. Single white arrow pointing at position of the subclavian artery (behind rib cage). The inferior cardiac branch was not used in this study and is located below the subclavian artery. VN, vagus nerve; ST, sympathetic trunk; CB, superior cardiac branch; CCA, common carotid artery.

For dissection, cadavers were placed in the dorsal recumbence position. A ventral skin incision (15–20 cm in pigs, 7–9 cm in rabbits) was cut on both sides next to the trachea starting at the level of the mandibles and extended further to the sternal notch. Tissue was dissected and the carotid sheath was opened to expose the common carotid artery, internal jugular vein, and the VN as well as the ST. Nerve dissection was started at the level of the nodose ganglion (NG) (Figure 1, “position.1”) and extended caudally to the superior cardiac branch (Figure 1 **“position 4”**), which originates approximately 2–5 cm cranial to the subclavian artery on both sides. The inferior cardiac branches were not dissected and included into this study since their anatomical branching point within the epicardial fat pad is not practically suitable for selective cardiac VNS. Branches emerging from and terminating in the VN were tracked to their origin structures. Both recurrent laryngeal nerves were identified as running close to the trachea and looping back around the subclavian arteries. Subsequently, the chest was opened by median sternotomy followed by gentle exposure of the heart. Then, the surgical preparation of the VN was continued towards the heart, and the superior CBs (one CB per nerve) were dissected starting from the cardiac branching points at the VN towards the insertion points at the heart. Care was taken not to cut any of the cross-connections between the VN and the ST, also referred to in this study, as “communicating branches”. The macroscopic branching and merging positions between the VN, ST, and superior cardiac branching were highlighted *in-situ* (Figures 2A,B). Throughout dissection, isotonic sodium chloride solution (NaCl, 0.9%) was used to prevent the nerves from drying out*.* Finally, the dimensions of the VN, from the nodose ganglion down to the insertion points of the cardiac branches into the heart were measured using a ruler and captured in a photo Figures 2A,B. In the following, the superior CB will be briefly named “CB”.

### 2.2 Vagal nerve preparation and harvesting

To maintain the anatomical *in-situ* orientations of the nerves after dissection and harvesting, surgical sutures (5–0 silk suture, Silkam^®^ B-Braun Aesculap, Tutlingen, Germany), were fixed onto the epineurium of the VN labeling the ventral side starting just below the nodose ganglion.

After complete dissection, nerves were placed and fixed onto a polystyrene plate with V-shaped incisions labeling the medial site, cut edges labeling the caudal site, and the sutures labeling the ventral sites of the VN, thus maintaining the anatomical *in-situ* orientation of the nerve specimen. Nerve specimens were briefly washed in PBS followed by fixation in 4% phosphate-buffered 4% PFA (Roti-Histofix, Roth, Karlsruhe, Germany) at 4°C for 16–18 h in case of pig nerves, and 14 h for rabbit nerves, respectively. After fixation, nerve samples were briefly washed again and stored in freshly made PBS at 4°C until staining for µCT imaging.

### 2.3 Nerve imaging and data analysis

#### 2.3.1 Pre-processing of native images

In order to provide comparable images of the native nerve specimen to the scanned versions on the identical nerves, native images showing the whole nerve specimen were preprocessed and anatomically labeled as shown in Figures 2A,B using the GIMP open-source software (GIMP 2.10.24, revision 2).

### 2.4 Micro-computed tomography

For contrast enhancement of the different types of tissue, nerves were stained in 14 ml 0.5% (rabbit nerves) or- 1% (pig nerves) Lugol’s solution for 24 h. Lugol’s solution is one part iodide and two parts potassium iodide in an aqueous solution. To produce a concentration of 1%, potassium iodide is dissolved in double-distilled water as described in a previous study [(Heimel et al., 2019)]. μCT scans were performed using a SCANCO μCT 50 (SCANCO Medical AG, Brutistellen, Switzerland) specimen μCT scanner. To avoid movement artifacts nerve specimens were placed in 15 ml conical centrifuge tubes in the residual staining solution with the polystyrene plate used for fixation. For overview scans low-resolution scanning was performed with 70 kVp (200μA, 0.5 mm Al filter, 550 projections, 100 ms integration time) at an isotropic resolution of 20 μm. The VNs from the pigs were divided into two nerve specimens of 7 ± 2 cm long half-sections, followed by digital alignment between scans over the entire length of the nerves (= cranial and caudal nerve specimen). Anatomical orientation for proper alignment of the two parts was given by a ventral suture that was placed to the ventral site of the nerve and a polystyrene plate on which the nerve was placed onto with its dorsal side. The medial site was labeled by the site where the cardiac branch left the VN trunk. The shorter rabbit nerves were scanned over the total length with 7 ± 1.3 cm of length and also the polystyrene plate labelling the dorsal site and the CB on the medial site. Therefore, it did not need any alignment of the scans afterwards.

High-resolution scans of both species were performed 2 days after the low-resolution scans at the site of the cardiac branching point with 70 kVp (85 μA, 0.5 mm Al filter, 850 projections, 350 ms integration time) at an isotropic resolution of 8.9 μm over 1 cm of nerve length. To maintain full saturation of fascicles with Lugol’s staining solution, nerves were re-stained 24 h prior to performing high-resolution scans.

High-resolution data were analyzed at the cardiac branching point and investigated whether a greater number of cardiac fascicles could be detected when higher resolution was applied to the nerves

### 2.5 Computerized 3D rendering and tracking of the cardiac branch

Raw images from µCT scans were provided as DICOM and tiff formats while only the DICOM format was exported for contrast-based segmentation of VN using Materialize Mimics Research 23.0 software (Materialize, Leuven, Belgium). The fascicles of the VN, the ST, and the CB were manually segmented and isolated, owing to partly low contrast between nerve and connective tissue, from the complete VN using split function to trace the course of the CB fascicles within the cervical VN from the CB point to the isolated cardiac branch. Particular focus was placed on the identification of the position where the fascicles of the CB traveled topographically in the cervical VN and whether they crossed or joined other fascicles. The STL file generated was then imported to Materialize 3-Matic 15.0 software (Materialize, Leuven, Belgium) for performing a remesh, necessary for reducing the size of the file by keeping the quality of the data basically unchanged. Here the connective tissue was further separated from the VN and CB. The segmented 3D models will provide data for subsequent elaboration e.g., simulation of electrical-field spatial distributions during stimulation.

### 2.6 Data analysis

All µCT data sets were available as tiff format and as dicom format. Firstly, µCT imaging stacks and single cross-sections were analyzed as TIFF files in XY, YZ, and XZ direction using Fiji software [(Schindelin et al., 2012), (Bergmeister et al., 2017)] for delineation, tracing and quantification (e.g., areas, fascicle counts). Firstly, each stack was rotated and aligned with the dorsal side of the nerves being on the upper part of the images. The dorsal and the medial sites were then labeled in each stack. For digital alignment of the whole pig nerves, both stacks (stack of the cranial and caudal part of the nerve) were opened in Fiji and the positions of nerve the fascicles on the last slides of the cranial part and those on the first slides of the caudal part were compared. The positions of the cardiac branch were labeled on the last slides of the cranial part and the first slides of the caudal part to maintain anatomical orientation. The contrast of the nerve cross-sections was manually enhanced for proper quantification of nerve fascicles using the brightness/contrast function based on tissue-dependent differences in gray values of nerve fascicles and background. Tracing of the fascicles of the cardiac branch was performed using the lasso tool in XY and XZ views. This was done in both species, pigs and rabbits. Here, the course of the cardiac branch fascicles was tracked from the cross-slice position of the CB point **(position 4,** Figure 1) up to the cervical VN, where the cardiac fascicles started either merging, splitting, or both with the cervical VN fascicles so that it was not possible to further distinguish them as single cardiac branch. This cross-slice position was labeled and the distance Figure 5A from the CB point up to this position was measured for each nerve (Figure 5A). The same procedure was then performed for mapping the distance of the first fascicle exchange position between the ST and the VN cranial to the cardiac branching point.

Next, we compared the findings regarding branching and merging patterns in µCT scans vs. macroscopically *in-situ*, out of which the following types could be differentiated in the pigs. The first type noted was **“contact”**, describing the situation where two nerves were closely adjacent to each other albeit there was still epineurium present between the nerves, thus no exchange of fascicles occurred. In some nerves, an additional layer of epineurium was surrounding the two nerves together. The next definition used in this study was **“merged”** fascicles representing two fascicles from each nerve fusing together and forming one fascicle surrounded by one layer of perineurium that further moved cranially as one fascicle, which was found between fascicles of the VN and ST at the mid-cervical level and between the VN and CB at the lower cervical level, from where fascicles of the CB merged with the fascicles of the cervical VN, thus, were not further distinguishable and traceable as “isolated” CB fascicles in the µCT scans. The third pattern, **“communicating” branches”**, also referred to as cross-connections, was observed between VN and ST at the mid-cervical level, where single nerve branches crossed the surgical field from one nerve to the other.

The next steps of image analysis of the functional anatomy were conducted only in the multifascicular nerves of pigs using also Fiji software (Schindelin et al., 2012). The area of the nerves and fascicle number was measured for each nerve at the cross-slice positions of interest using the freehand selection tool followed by manual counts of the fascicle numbers using the multipoint tool and the freehand tool. Measurements on cross-sections were performed at five different positions as shown in Figure 1, starting from just below the nodose ganglion (position 1) down to just below the cardiac branch (position 5). Positions 2 and 3 were located at the mid-cervical level approximately 2 cm apart from each other. Position 4 was the position of the cardiac branching point (CB point).

### 2.7 Statistical analysis

Data were analyzed using Graphpad Prism software (GraphPad Software, San Diego, CA, United States) and MATLAB (version R2021a, Mathworks, Natick, Massachusetts, United States).

Topographical measurements of the cardiac branching patterns were analyzed using descriptive statistics and are presented as absolute and relative values.

Normal distribution of data was assessed by the Shapiro-Wilk test. Non-normally distributed data is presented as median and interquartile range (IQR). Side-related comparisons were performed using the Kruskal–Wallis test for the variables fascicle numbers and area at level of the CB point. Statistical significance was accepted if *p* < 0.05.

## 3 Results

### 3.1 Surgical dissection and macroscopic examination of nerve samples

Dissections of the VN and the superior cardiac branch revealed that the branching position of the superior CB (“position 4”, Figure 1) varied among individuals in pigs and between left and right side. However, we could find the branching position of the superior cardiac branch to be at 2–5 cm cranial to the subclavian artery. In rabbits, the superior cardiac branch was found to branch off closer (approximately 1–3 cm) to the level of the subclavian artery in all animals.

To determine whether VN and ST use separate nerve fibers or, conversely, share them in order to send signals from the cervical level downwards to the heart and other organs, surgical dissection and macroscopic examinations were applied to fresh swine cadavers as well as those of rabbits.

The results of the surgical dissection in pigs revealed different merging and branching patterns among individuals (Table 1). Close contact between or even merging between VN and ST was macroscopically observed in 7/11 (64%) nerves, of which 3/7 (43%) nerves were on the left side, and 4/7 (57%) nerves were to the right. In contrast, when it came to the rabbit nerves, no prominent differences in branching patterns were observed and no difference was determined between the left and right sides. Hence, unlike the situation in pigs, in rabbits, the fascicles in the VN and ST were clearly separated from each other in the cardiac branches towards the heart, and it would be interesting to gain further insight into the situation in pigs using more advanced tomography.

**TABLE 1:** Overview of the branching and merging patterns between VN, ST, and CB in µCT data of pig nerves. Numbers are presented as absolute and relative values. The first pattern, “contact”, with epineurium between VN and ST is depicted in **column 1)**. The second pattern “merging fascicles” is represented in **columns 2A and 2B** and presents the numbers of nerves, in which merging of fascicles between the VN and the ST (column 2A), and the VN and the CB (column 2B), respectively, occurred. **Column 3** represents the third pattern “communicating branches between ST and VN”, which are cross-connections between these two nerves that were found in both preparations at the cervical level, the macroscopic dissections, and µCT data. **Column 4** depicts the number of observations during dissections, where the VN and the ST nerves were showed either close contact or merging interactions (“merging or contact”) with each other *in-situ*.VN, Vagus Nerve; CB, cardiac branch; ST, Sympathetic trunk.

Nerves	Counts total n [%]	1) Total VN/ST contact in µCT [n nerves]	2A) Total VN/ST merging fascicles in µCT [n nerves]	2B) Total VN/CB merge fascicles in µCT [n nerves]	3) Communicating branches ST/VN total [n nerves]	4) VN/ST merging or contact total macroscopic [n nerves]
Left side	5 [45%]	3 [27%]	1 [9%]	3 [38%]	2 [18%]	3 [43%]
Right side	6 [55%]	4 [36%]	2 [18%]	5 [45%]	4 [36%]	4 [57%]
Total	11 [100%]	7 [64%]	3 [27%]	8 [73%]	6 [55%]	7 [64%]

### 3.2 µCT scans in low- and high-resolution of the cardiac branching points in pigs

To investigate whether fascicle number in the cardiac branches (CBs) differed between the two scanning protocols low-resolution vs. high-resolution scans, all nerve specimens were firstly scanned in low-resolution (pig: n = 11; rabbit: n = 5) over the entire length (pigs: 7 ± 2 cm, rabbits 7 ± 1.3 cm) followed by high-resolution scans over 16.906 ± 2.803 mm at level of the cardiac branching point.

In both scans fascicles were clearly separated from the rest of the nerve tissue, nevertheless, boundaries between single fascicles were better visible in high-resolution scans.

**FIGURE 3:**
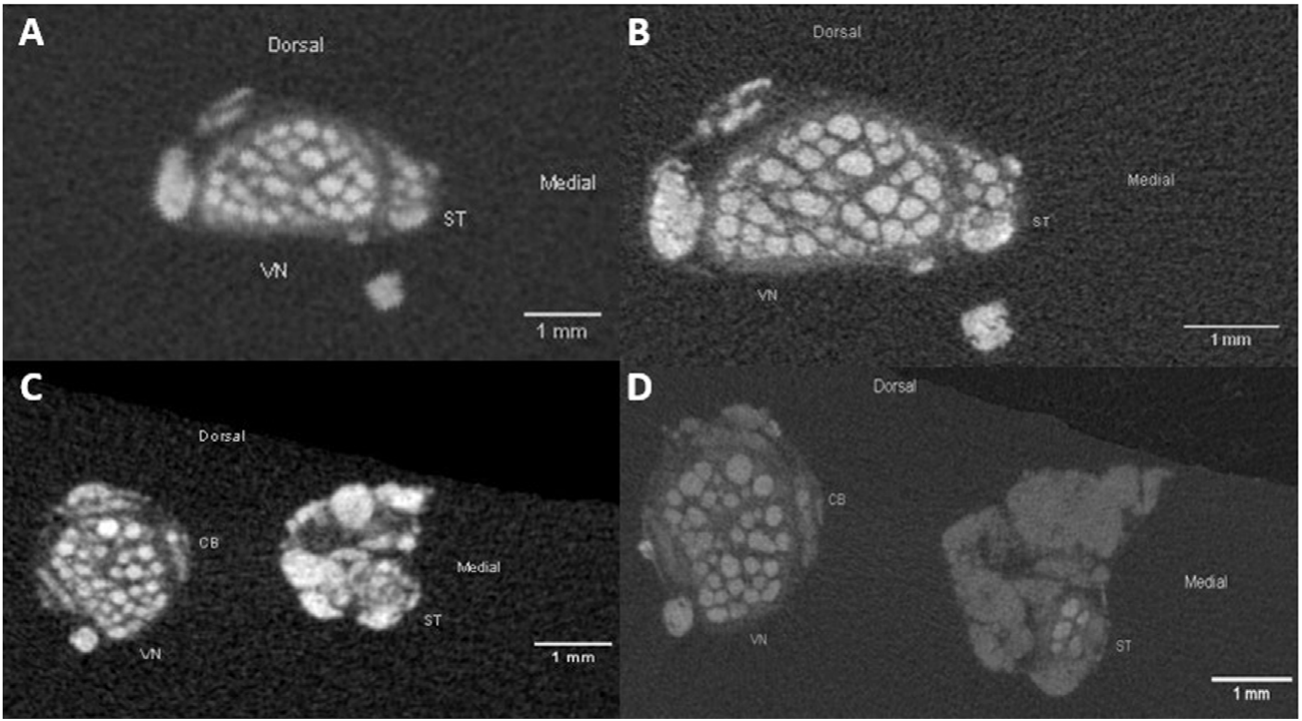
Comparison of the pig VN and ST anatomy in cervical nerve specimen in low-resolution **(A)**; 20 µm isotropic resolution and high-resolution **(B)**; 8.9 µm isotropic resolution). Differences are visible in the sharper representation of structures of interest, such as fascicles and epineurium. The silk sutures fixed on the ventral side of the nerves are represented by the bright spots on the bottom site of the images. **(C,D)**: Example images of a caudal nerve specimen showing the anatomical comparison of the VN, ST and CB at level of the cardiac branching point in low-resolution (panel C; 20 µm isotropic resolution) and high-resolution (panel D; 8.9 µm isotropic resolution). VN, cervical vagus nerve; ST, sympathetic trunk; CB, cardiac branch.

The direct comparison between the low-resolution and high-resolution scans revealed a better distinguishability of single fascicles in HR scans, but it did not change the detectable number of cardiac fascicles at the level of the CB point (Figure 3).

### 3.3 3D rendering of µCT data and modeling selective cardiac VNS in an organism

**FIGURE 4:**
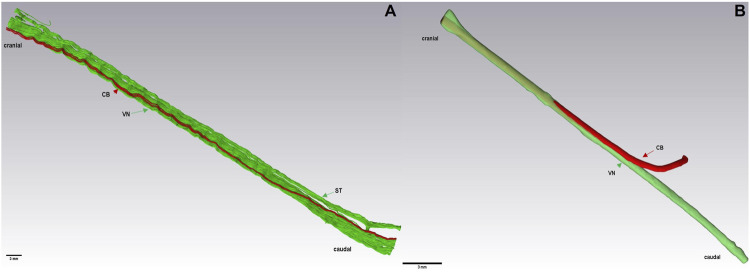
3D rendering of a caudal nerve specimen (7 cm) of the VN, ST, and CB at the level of the cardiac branching point in a pig **(A)** and of the VN and CB in a rabbit **(B)**, respectively. Here, the ST travels closely adjacent to the VN. Green: fascicles of the VN and ST. Red: cardiac branch fascicle. VN, Vagus Nerve; CB, cardiac branch; ST, Sympathetic trunk.

To understand further the anatomical structure of the cardiac autonomic innervation in both species, µCT scans were also 3D-rendered and an anatomical model of both nerves was developed. Data have shown that the 3D rendering of the multi-fascicular anatomy of the pig VN (Figure 4A) and the mono-fascicular anatomy of the rabbit VN (Figure 4B). In both species, the cardiac branch (indicated in red) enters VN (indicated in green) on the medial side, then travels in ventromedial position in the cervical VN towards cranial adjacent to other fascicles until it merges with other fascicles, as depicted in Figure 4.

### 3.4 Branching and merging patterns in pig nerves under µCT data examination and *in-situ*

Table 1 shows the three types of branching patterns we identified in this study. Therefore, the following patterns were defined: 1) contact between VN and ST, 2A) merged fascicles between VN and ST and 2B) merged fascicles between VN and CB fascicles, and 3) cross-connections between VN and ST, referred to as “communicating branches”. In addition, close contact or merging of VN fascicles with ST was macroscopically investigated during surgical dissections (Table 1 **, column 4).**

Several features were noted, as follows (Table 1): 1) Tight contact between the VN and ST was observed in 7/11 (64%) nerves, with 3/11 (27%) nerves on the left and 4/11 (36%) nerves on the right side. 2A) Merging fascicles, VN and ST were observed in 3/11 (27%) nerves, with 1/11 (9%) observations on the left side and 2/11 (18%) observations on the right side, respectively. 2B) Merging fascicles between VN and CB were observed in 10/11 (90%) nerves, of which 4/10 (40%) nerves were located on the left and 6/10 (60%) nerves on the right side. 3) In 6/11 (55%) of nerves communicating branches were found, of which 2/11 (18%) nerves were located on the left and 4/11 (36%) on the right side. 4) Macroscopical investigations of the branching- and merging patterns *in-situ* revealed 7/11 (64%) observations, with 3/7 (43%) on the left and 4/7 (57%) on the right side. A comparison of the branching patterns between the left and the right side displayed slightly more branching patterns on the right side.

### 3.5 Traceability of the cardiac fascicles in the vagus nerve of pigs and rabbits

**FIGURE 5:**
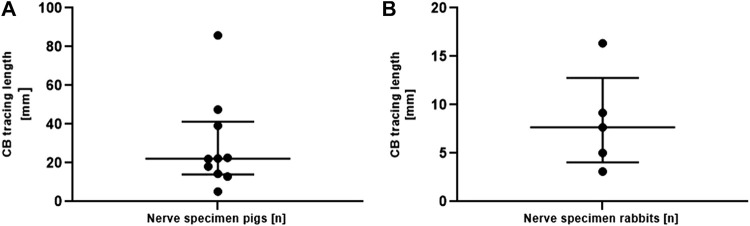
**(A)**. Length measurements of the traceability of the isolated superior CB in the vagus nerve of pigs (panel A) starting from the cardiac branching point. Data are depicted for each individual nerve. Measurements are given as median and interquartile ranges. Note the different scaling of the *y*-axis in both graphs. CB, cardiac branch. **(B)**. Length measurements of the traceability of the isolated superior CB in the vagus nerve of rabbits starting from the cardiac branching point. Data are depicted for each individual nerve. Measurements are given as median and interquartile ranges. Note the different scaling of the *y*-axis in both graphs. CB, cardiac branch.

To measure the traceable length of the fascicles of the cardiac branch in the cervical VN trunk in both species, the cardiac branch was traced starting from the cardiac branching point in cranial direction (Figure 5B **)** In total, the cardiac branch could be traced cranially in 10/11 (90.9%) of pig nerves and in all rabbit nerves (5/5). The median traceable length of the pig cardiac branch was 22.19 mm (IQR 14.02–41.30 mm) and 7.68 mm in rabbits (IQR 4.06–12.77 mm).

### 3.6 Variations in fascicle exchange above the cardiac branching point in pig nerves

**FIGURE 6:**
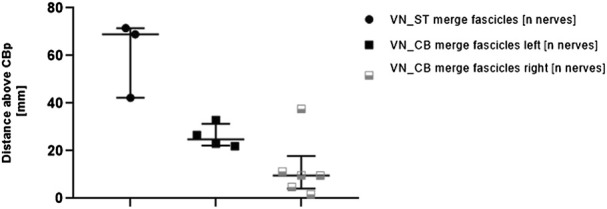
Comparisons of distances measured for the first fascicle exchange above the cardiac branching point between 1) VN and ST (left column, VN_ST) and 2) VN and CB (middle and right column, VN_CB merge fascicles left/right). Each point or square represents one nerve. The distances between the corresponding fascicle merging positions and the cardiac branching points were measured in the µCT stacks. Since more fascicle-merging events were counted for the VN_CB, they were also compared between the left and the right side. Data are represented as median and interquartile range. CBp, cardiac branching point; VN, vagus nerve; ST, sympathetic trunk.

To explore differences in merging and branching patterns of fascicles between ST and VN in pig nerves, fascicle-merging events were counted manually in individual nerves between VN and ST using the µCT scans (Figure 6).

Merging of fascicles between VN and CB (VN_CB fascicles) were visible in 10/11 (90.0%) nerves. Out of these 10 nerves, 4/10 (40%) observations were located on the left side, and right 6/10 (60%) observations on the right side. In contrast, merging of single fascicles between VN and ST (VN_ST fascicles) on the upper cervical level was counted in 3/11 (27%) animals. The median distance of the fascicle merging positions between VN and ST was 68.98 mm (IQR 42.32–71.54 mm) above the cardiac branching point. The median distance for merging of fascicles between VN and CB was 24.82 mm (IQR 22.24–31.37 mm) on the left side and 9.66 mm (IQR 4.09–17.86 mm) on the right side. The calculated *p*-value for the group comparison left vs. right side “merging fascicles” was not significant (*p* = 0.361).

### 3.7 Comparisons of nerve size and fascicle numbers in µCT scans at different anatomical positions

To examine whether there was a correlation between fascicle numbers and anatomical positions of the VN, the median values for the two features were measured for the VN, ST, and CB point at different positions (Figure 1) and are listed in Table 2.

**TABLE 2:** Measurements of the nerve areas (Area) and fascicle numbers (Fascicle number) at multiple locations along the cervical level down to just caudal to the cardiac branching point across the cohort. Lacking measurements at positions where the cor corresponding nerve was not present or measurable were designated “NaN” (“not a number”). Numbers are represented as median and interquartile range (IQR). Area measurements are given in [mm^2^], fascicle numbers in total numbers [n]. The minimum (Min) and maximum (Max) values of the measurements were determined for each nerve.VN, vaus nerve; ST, sympathetic trunk; CB, cardiac branch.

Cross-slice position	Area VN [mm^2^]	Area ST [mm^2^]	Area CB [mm^2^]	Fascicle number VN [n]	Fascicle number ST [n]	Fascicle number CB [n]
Position 1	2.98	0.51	NaN	24	7	NaN
Position 2	2.82	0.52	NaN	26	5	3
Position 3	1.94	0.63	NaN	19	6	1
Position 4	2.00	0.38	0.23	26	4	4
Position 5	2.29	0.63	0.18	24	8	NaN
Median total	2.29	0.52	0.23	24.00	5.75	3.00
IQR total	0.56	0.06	0.16	0.00	0.38	1.25
Max	2.98	0.63	0.23	26	8	4
Min	1.94	0.38	0.18	19	4	1

**FIGURE 7:**
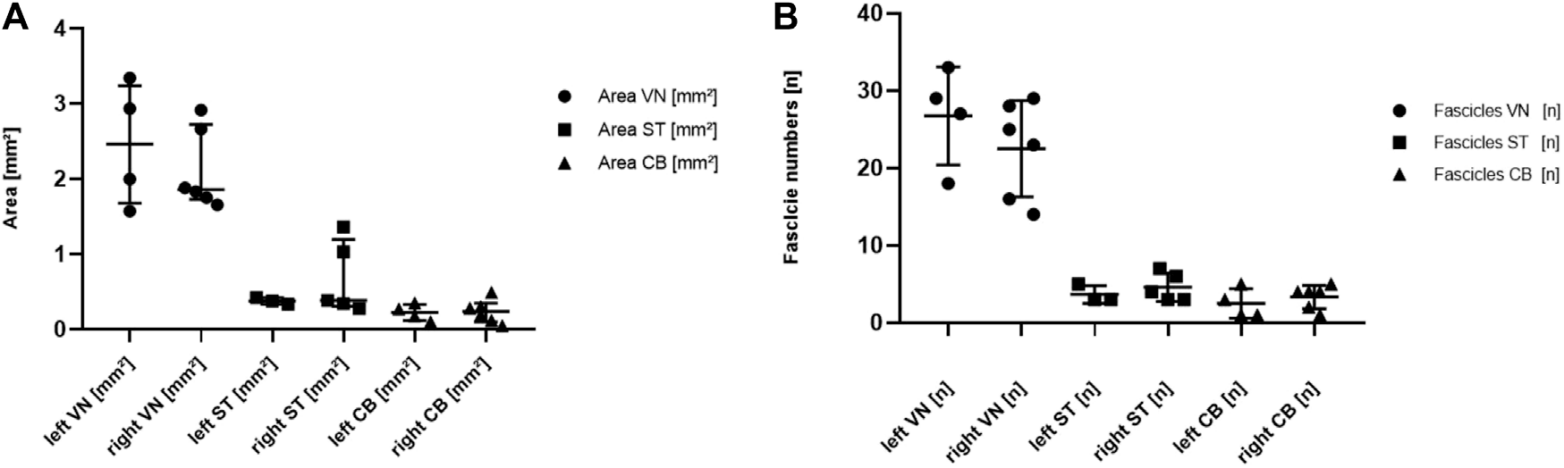
**(A)**. Comparison of the nerve area between VN, ST, and CB at the level of the cardiac branching point. Each dot represents one nerve. Data are represented as median and interquartile range. VN, vagus nerve; ST, sympathetic trunk; CB, cardiac branch. **(B)**.Comparison of the fascicle numbers in the VN, ST, and CB at the level of the cardiac branching point for the left (left columns) and the right (right columns) sides. Each dot represents one nerve. Data are represented as median and interquartile range. VN, vagus nerve; ST, sympathetic trunk; CB, cardiac branch.

The comparisons at different levels show that the widest median area of the cervical VN was found at position 1, and at position 3 in the ST. The smallest median area of the VN was at position 3 and that of the ST was at position 4. The largest median area of the cardiac branch was found just at the cardiac branching point (pos.4). The measurements for these comparisons taken at the level of the cardiac branching point (pos. 4) are shown in Figures 7A,B, presenting the side-related comparisons of the median values of the parameters “fascicle numbers” and “nerve area” at the level of the CB point. The median area of the left and right VN was 2.47 mm^2^ (IQR 1,7 to 3.2 mm^2^) and 1.86 mm^2^ (IQR 1.2–2.7 mm^2^), of the left ST 0.38 mm^2^ (IQR 0.3–0.4 mm^2^), and right 0.38 mm^2^ (IQR 0.3–1.2 mm^2^), and of the left vs. right CB 0.22 mm^2^ (0.1–0.3 mm^2^) and 0.24 mm^2^ (0.1–0.3 mm^2^). The calculated *p*-values for side comparisons in each of the three nerves in terms of the area were not statistically significant (VN *p* = 0.39; ST *p* = 0.46; CB *p* = 0.67). The median values of VN counted fascicles at the level of the CB point were n = 28 on the left side (IQR 20 to 32 fascicles) and n = 24 fascicles on the right side (IQR 16 to 28 fascicles), in the ST n = 3 (IQR 3 to 5 fascicles) on the left and n = 4 fascicles (IQR 3 to 7 fascicles) on the right side, respectively and in the CB n = 2 (IQR 1 to 5 fascicles) on the left and n = 4 (IQR 2 to 4 fascicles) on the right side. The calculated *p*-values for side comparisons in each of the three nerves in terms of the fascicle distributions were not statistically significant (VN *p* = 0.24; ST *p* = 0.43; CB *p* = 0.44).

## 4 Discussion

This study was aimed at mapping the anatomy and topography of the superior cardiac branches of the vagus nerve in two animal species typically used for neurostimulation studies. Accordingly, surgical dissections were performed in pigs and rabbits, and a species-comparative imaging approach was established using µCT and 3D rendering to map the cardiac autonomic innervation with emphasis on the cardiac vagal branches from the level of the nodose ganglion down to the cardiac branches of VN.

Surgical dissections revealed differences between the two species. In rabbits, the ST (medial) and the cervical VN (lateral) were running separately from each other towards caudal. No communicating branches between the two nerves were visible. Consequently, the cardiac VN branches terminated forming the cardiac plexus separately from ST branches facilitating an approach for selective cardiac VN stimulations.

In contrast, the surgical dissections in pigs have shown few cross-connections between the VN and the ST in the cervical region in some individuals. In addition, it was found that the ST was running medially to the cervical VN or looping in and out of the cervical VN among individual nerves. Hence, in three nerves, the cardiac branches might be composed of both autonomic qualities, from the VN and the ST whereas in the other individuals, the cardiac branches were only composed of vagal fibers. Pigs are usually considered as being the most comparable animal models for human VN anatomy [(Hammer et al., 2018; Stakenborg et al., 2020)], whereas rabbits are most similar to humans in terms of electrophysiology and curve of action potentials compared to other animal models [(Pohl et al., 2016; Quinn and Kohl, 2016; Fabbri et al., 2017)].

Using microcomputed tomography imaging and subsequent 3D rendering, we revealed here different merging and branching patterns of the cardiac branches among species. µCT is a non-destructive, time-efficient imaging technique, allowing for neuronal mapping of long nerve sections with a resolution suitable for tracing nerve fascicles and investigating nerve morphology on cross-sections [(Thompson et al., 2020a; Heimel et al., 2019; Yan et al., 2017; Thompson et al., 2020b; Ravagli et al., 2020; Ravagli et al., 2021)]. Comparisons of the surgical dissections and µCT imaging data revealed that µCT low-resolution scans provide additional information about the fascicular anatomy of the cardiac branch as well as the branching and fascicle exchange patterns between the autonomic nerves up to 15 cm length of nerve. With respect to the morphological information concerning the number, size, and branching patterns of the nerve fascicles, the high-resolution scans taken at the level of the cardiac branching points did not provide additional anatomical information about the cardiac fascicles compared to the low-resolution images, but it provided a sharper representation of fascicles which facilitates segmentation procedures. Therefore, further validation and studies of the anatomical features in the cardiac branch, such as epineural and perineural thickness, nerve fibers quantifications, and cellular structures need to be investigated using imaging techniques capable of applying even higher resolutions. This would provide additional information to the images, as shown in histology [(Thompson et al., 2020a; Hammer et al., 2018; Settell et al., 2020)] and high-resolution episcopic microscopy (HREM) [(Geyer et al., 2017; Dabiri et al., 2020; Keuenhof et al., 2021)], in combination with immunohistochemistry [(Gesslbauer et al., 2017)]. In addition to the µCT data, tracing of the cardiac branches in our 3D model provided further information mimicking the anatomy and topography of the cardiac vagal innervation *in-situ*.

As previously described in the methods section, our findings revealed three different types of branching and merging patterns between the VN, ST and CB nerve trunks as well as their fascicles, namely “contact”, “merged” fascicles, and “communicating” branches, also referred to as “cross-connections”. The latter were also described in the study by Settell et al. (2020) and could be confirmed by our present study.

We could find each of these patterns in the pig nerves, but not in the rabbit nerves. This further raises firstly the question, whether or how many of these patterns can be found in each individual pig used for selective VNS, and secondly, since individual differences in pigs appeared to exist, what the situation looks like in each individuum. This finally might impact the fascicle composition of the cardiac VN branch, which is important to be considered for studies aiming at selective cardiac VNS. All in all, the anatomical interactions between the autonomic nerves in pigs challenge the question, which fiber types, qualitatively and quantitatively as well as cholinergic or adrenergic, form the corresponding CB. In order to get an overview of the branching patterns during surgery, the two autonomic nerves should be parted from NG down to CB during surgical dissections, thereby providing insight into the optimal electrode positioning by the neurosurgeons.

In addition to the branching and merging patterns, the traceability of the isolated CB was measured using µCT scans in pigs and rabbits starting from the CB point up to the cervical VN. In both species, CB first remained on the medial side where it branched off the VN, and then migrated towards the ventro-medial site the further it was traced cranially, which was also confirmed by the digital 3D models. These findings underlined the hypothesis of a organotopic organization of fascicles [(Thompson et al., 2020a; Settell et al., 2020; Thompson et al., 2022)], which would help map the functional anatomy of CB.

In the next step, we analyzed the functional anatomy of nerve fascicles of VN, ST, and CB for the two anatomical features “nerve area” and “fascicle numbers” that were performed at the level of the CB point **(“position 4”,** Table 1 **).** We found that cardiac fascicles were completely separated from the other fascicles and started merging from 7.68 mm in rabbits, and 22.19 mm in pigs above the CB point. Therefore, our data suggest that the most optimal position for selective cardiac VNS proposed to be just above the CB point, where cardiac fascicles start separating from the other fascicles in the vagal trunk. The data of this study showed that the cardiac branches travelled on the edge position just above the CB point, which facilitated the selective neuromodulation of the cardiac vagal fibers.

With respect to translational medicine and application of selective cardiac VNS in humans, previous studies have shown that the pig is the most representative and comparable animal model for anatomical studies, both in terms of size and shape of VN, albeit the number and composition of fascicles differs greatly between the two species [(Hammer et al., 2018; Verlinden et al., 2016)]. This may cause some difficulty in the development and subsequent evaluation of neuro-electrodes and stimulation strategies intended for human applications [(Settell et al., 2020)].

The importance of anatomical- and species-related considerations for selective VNS has also been shown in other studies demonstrating that the nerve fiber composition of VN changes depending on species and topographical positions (30, (Stakenborg et al., 2020; Verlinden et al., 2016; Hammer et al., 2015)). Hence, the anatomical differences of the CB compositions observed in this study, as well as the topographical position of the stimulated structures will determine the outcome of approaches used in selective cardiac neuromodulations. In addition, other approaches for selective neuromodulation, such as detection of target nerve fascicles using electrical impedance tomography or multielectrode arrays stimulation [(Ravagli et al., 2020; Ravagli et al., 2021; Plachta et al., 2013; Aristovich et al., 2021; Fitchett et al., 2021)] will greatly benefit by the anatomical knowledge of micro-fascicular arrangements in the autonomic nerves. Taken together, a better anatomical understanding of the cardiac autonomic innervation will help surgeons and engineers to optimize the intraoperative placement and refinement of selective cardiac electrodes.

## 5 Limitations of the study

Surgical dissections, µCT imaging and 3D renderings were used in this study since they provide an anatomical map of the course and connections of the cardiac innervation on a fascicular level over several centimeters of nerve. However, these methods do not allow anatomical identification and quantification of nerve fibers but not specific types of nerve fibers, but they do allow studying the overall cardiac innervation and the fascicle anatomy in pigs and rabbits. This emerged as an important factor to be considered in terms of translational medicine and planning of future cardiac VNS studies in humans and animals.

In addition, the outcome of image segmentations of µCT data and 3D rendering depend on the software capabilities and contrast-staining of tissues. Our high-resolution scans appear partly brighter than the low-resolution scans, since they were performed 2 days after the low-resolution scans and thus, had to be re-stained for 24 h before re-imaging.

Therefore, we are working on further studies where we use h imaging techniques with higher resolutions capable of providing additional information, such as high-resolution episcopic microscopy and immunohistochemistry to classify further the anatomy of the cardiac vagal innervation. In addition, to understand fully the functional organization of VN at the cervical level, in which cardiac fibers are also travelling, selective stimulations of afferent fibers from the visceral organs as shown in [(Thompson et al., 2019; Thompson et al., 2020b)] might be a helpful approach in future studies.

## 6 Conclusion

In this study, we mapped the course of the cardiac vagal branches starting from the CB point up to the cervical VN, as well as the topographical interaction between VN and ST. In addition, the functional anatomy of CB, VN, and ST was further investigated at the level of the CB point. Our findings revealed that CB travels from the medial side where it branches off to ventromedial position in VN, and that the number as well as size of nerve fascicles changed depending on the anatomical position. Therefore, we suggest that the best position for selective stimulation of pure cardiac VN fascicles right above the CB point, where the target fascicles travel on the edge position in VN, which makes them better accessible for selective cardiac stimulation than on the upper cervical level. Our data provided key findings on the species-related CBs and highlighted the need for further characterization of the cardiac autonomic innervation and novel in-silicio models to improve stimulation patterns for selective cardiac VNS.

## Data Availability

The original contributions presented in the study are included in the article/supplementary material, further inquiries can be directed to the corresponding authors.
