# Corrigendum: Mapping the functional anatomy and topography of the cardiac autonomic innervation for selective cardiac neuromodulation using MicroCT

**DOI:** 10.3389/fcell.2023.1193013

**Published:** 2023-03-31

**Authors:** Bettina Kronsteiner, Lydia M. Zopf, Patrick Heimel, Gunpreet Oberoi, Anne M. Kramer, Paul Slezak, Wolfgang J. Weninger, Bruno K. Podesser, Attila Kiss, Francesco Moscato

**Affiliations:** ^1^ Center for Medical Physics and Biomedical Engineering, Medical University of Vienna, Vienna, Vienna, Austria; ^2^ Ludwig Boltzmann Cluster for Cardiovascular Research, Vienna, Vienna, Austria; ^3^ AUVA Research Centre, Ludwig Boltzmann Institute for Experimental and Clinical Traumatology, Vienna, Vienna, Austria; ^4^ Austrian Cluster for Tissue Regeneration, Vienna, Vienna, Austria; ^5^ Karl Donath Laboratory for Hard Tissue and Biomaterial Research, University Clinic of Dentistry, Medical University of Vienna, Vienna, Vienna, Austria; ^6^ Department of Anatomy, Center for Anatomy and Cell Biology, Medical University of Vienna, Vienna, Vienna, Austria; ^7^ Division of Anatomy, Center for Anatomy and Cell Biology, Medical University of Vienna, Vienna, Vienna, Austria

**Keywords:** vagus nerve stimulation, cardiovascular diseases, fascicular anatomy, selective cardiac, neuromodulation, microcomputed tomography, 3D rendering

In the published article, there was an error in “Figure 1. Schematic overview of the surgical window in a pig” as published. The dashed line indicating the anatomical “position 4” was accidently moved to the wrong position. The correct position is right above the branching point of the vagal cardiac branch as correctly written in the main text. The corrected figure “Figure 1 Schematic overview of the surgical window in a pig” and its caption “Schematic overview of the surgical window in a pig. The VN can be distinguished from the ST by identification of the NG. In some individuals, the two nerves were observed to merge or split at the cervical level. In rabbits, the VN and the ST were travelling individually next to each other to the cardiac plexus. Five anatomical positions (pos 1-pos 5) were defined from cranial to caudal at which the fascicle number and nerve areas were measured. The superior CB was the branch used in this study (one superior CB per nerve) and harvested right above the level of the subclavian artery (pos.5); the inferior CB (indicated as dashed lines) was not used in this study since its anatomical position in the epicardial fat is surgically less feasible for selective stimulation than the superior CB. Pos. 1-3 label the upper to the mid-cervical level. Pos.4 indicates the cardiac branching point of the superior CB. NG, nodose ganglion; CCA, common carotid artery; VN, Vagus Nerve; ST, Sympathetic trunk; Pos, position; sup. cardiac branch, superior cardiac branch; inf. cardiac branch, inferior cardiac branch.” appear below.

**FIGURE 1:**
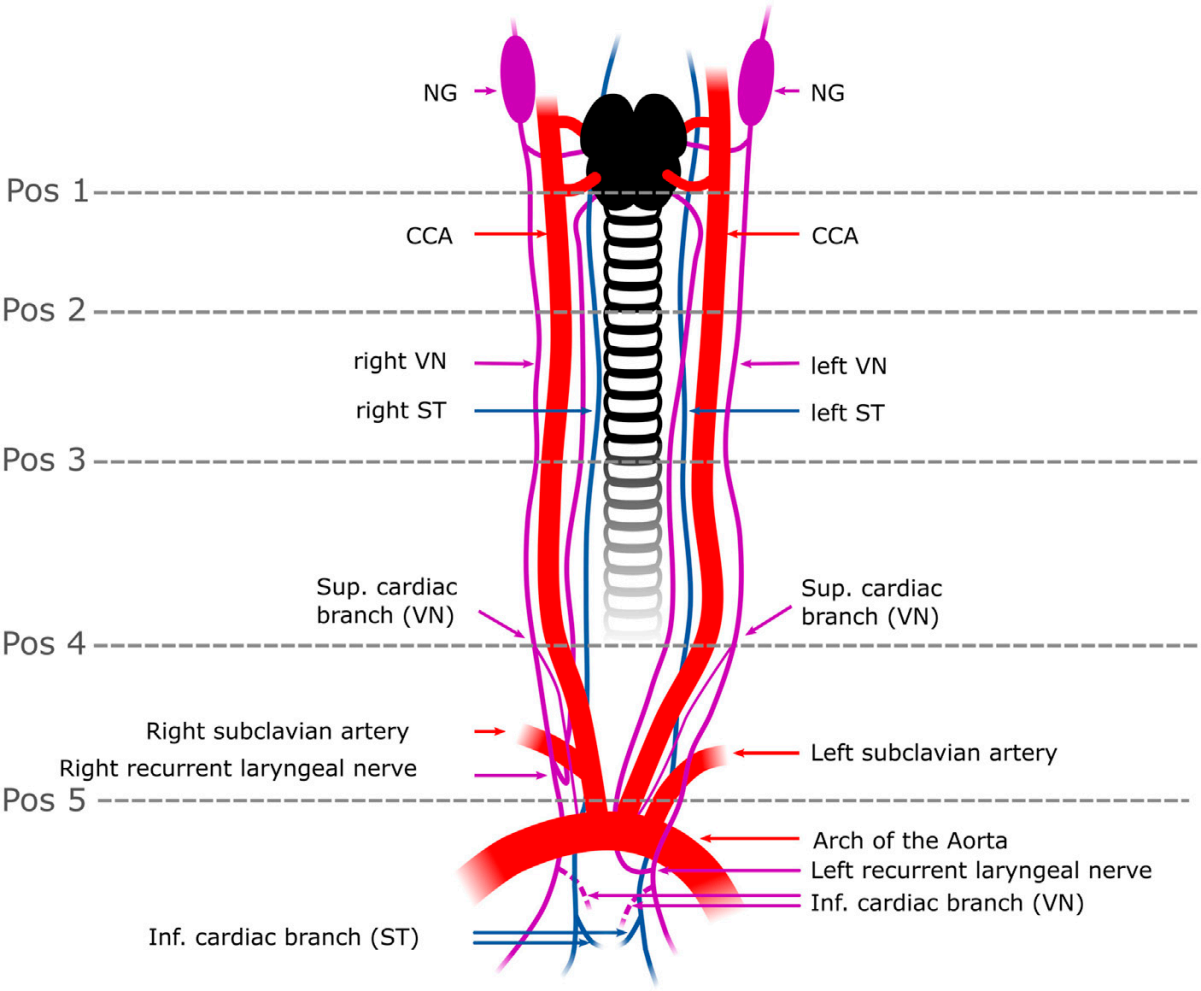
Schematic overview of the surgical window in a pig. The VN can be distinguished from the ST by identification of the NG. In some individuals, the two nerves were observed to merge or split at the cervical level. In rabbits, the VN and the ST were travelling individually next to each other to the cardiac plexus. Five anatomical positions (pos 1-pos 5) were defined from cranial to caudal at which the fascicle number and nerve areas were measured. The superior CB was the branch used in this study (one superior CB per nerve) and harvested right above the level of the subclavian artery (pos.5); the inferior CB (indicated as dashed lines) was not used in this study since its anatomical position in the epicardial fat is surgically less feasible for selective stimulation than the superior CB. Pos. 1-3 label the upper to the mid-cervical level. Pos. 4 indicates the cardiac branching point of the superior CB. NG, nodose ganglion; CCA, common carotid artery; VN, Vagus Nerve; ST, Sympathetic trunk; Pos, position; sup. cardiac branch, superior cardiac branch; inf. cardiac branch, inferior cardiac branch.

The authors apologize for this error and state that this does not change the scientific conclusions of the article in any way. The original article has been updated.

